# Potential of immunosuppressive agents in cerebral ischaemia

**Published:** 2011-01

**Authors:** Yogendra Kumar Gupta, Anjali Chauhan

**Affiliations:** *Department of Pharmacology, All India Institute of Medical Sciences, New Delhi, India*

**Keywords:** Cerebral ischaemia, immunosuppressants, *in vitro* and *in vivo* models, neuroprotection

## Abstract

Ischaemic stroke is a disorder involving multiple mechanisms of injury progression including activation of glutamate receptors, release of proinflammatory cytokines, nitric oxide (NO), free oxygen radicals and proteases. Presently, recombinant tissue plasminogen activator (rtPA) is the only drug approved for the management of acute ischaemic stroke. This drug, however, is associated with limitations like narrow therapeutic window and increased risk of intracranial haemorrhage. A large number of therapeutic agents have been tested including N-methly-D-aspartate (NMDA) receptor antagonist, calcium channel blockers and antioxidants for management of stroke, but none has provided significant neuroprotection in clinical trials. Therefore, searching for other potentially effective drugs for ischaemic stroke management becomes important. Immunosuppressive agents with their wide array of mechanisms have potential as neuroprotectants. Corticosteroids, immunophilin ligands, mycophenolate mofetil and minocycline have shown protective effect on neurons by their direct actions or attenuating toxic effects of mediators of inflammation. This review focuses on the current status of corticosteroids, cyclosporine A, FK506, rapamycin, mycophenolate mofetil and minocycline in the experimental models of cerebral ischaemia.

## Introduction

Cerebral stroke (brain attack) is the most life- threatening cerebovascular disorder, the second leading cause of death and principle cause of disability in the world^1^. Even with advances in treatment of stroke, 20-50 per cent of the patient die within a month or become dependent on others^2^. Stroke results due to interruption of cerebral blood flow causing irreversible and fatal damage to the affected neurons. There are two main types of strokes, ischaemic and haemorrhagic. Ischaemic stroke accounts nearly for 85 per cent of all reported stroke incidents and is the main focus of the current studies. This type of stroke occurs when a thrombus or embolus blocks cerebral blood flow resulting in cerebral ischaemia and consequently neuronal damage and cell death. Haemorrhagic stroke occurs due to rupture of any blood vessel in the brain resulting in rapid cerebral damage and accounts for the remaining 15 per cent stroke cases.

Intravenous recombinant tissue plasminogen activator (rtPA) is the only approved therapy for management of ischaemic stroke^3^. Patients who receive this drug within the initial 3 h therapeutic window also have a high risk of intracranial haemorrhage, usually 6-8 per cent against 0.6-2 per cent spontaneous hemorrhages in stroke^4^–^5^. Other limitations associated with rtPA therapy like disruption of blood brain barrier; seizures and progression of neuronal damage^6^–^8^ are major concerns. Thus, there is a continued need for exploring novel neuroprotective strategies for the management of ischaemic stroke.

Recent studies on immunosuppressive agents have revealed their neuroprotective potential in ischaemic stroke. Immunosuppressive agents have shown promise as being neuroprotective in safeguarding the neurons against excitotoxic insults and also improving neurological functions and infarct volume in experimental models of ischaemic stroke^9^–^13^. These agents have direct effect on microglia cells and inhibit mediators of inflammation. In order to appreciate the potential role of immunosuppressive agents in ischaemia, revisiting the pathophysiology of cerebral ischaemia is required. This review briefly focuses on the mechanisms involved in cerebral ischaemic stroke and how the immunosuppressive agents have shown potential in its management.

### The aetiopathology and mechanisms of cell death in ischaemia

The interruption in blood flow to the brain results in reduced supply of oxygen and nutrients to the neurons. The lack of blood supply results in two identifiable areas namely the core and penumbra. The core which is a neuronal dead area is not accessible to therapeutic intervention whereas the penumbra is a still salvageable zone and is the target of the most therapeutic interventions (Fig.). The consequence of ischaemia can briefly be described as below.

**Fig.**
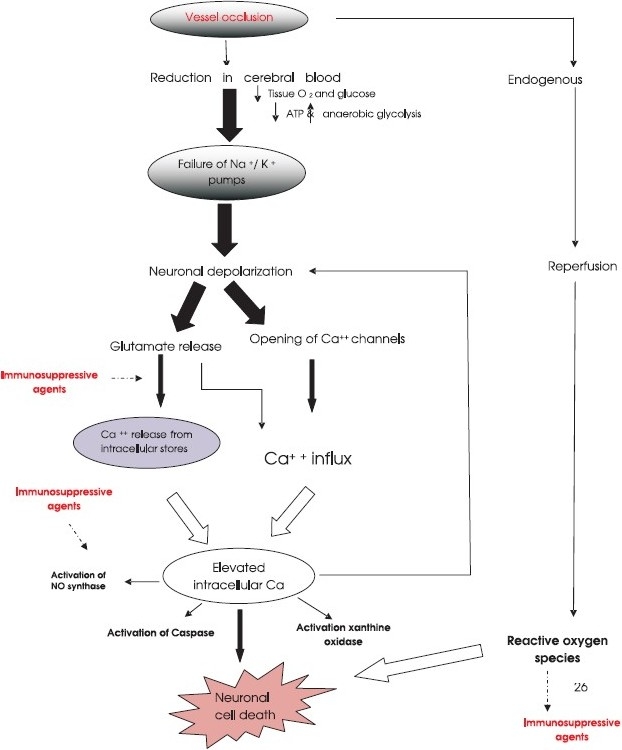
A simplistic presentation of the cascade of events occurring in cerebral ischaemia and possible sites of immunosuppressive agents actions.

**Energy depletion:** Consequent to reduction/loss of blood supply inside the core, the adenosine triphosphate (ATP) levels are reduced leading to incarceration of cellular metabolism^14^. The lack of energy results in impaired ion homeostasis.

**Calcium overload and activation of glutamate receptors:** Disrupted ion homeostasis leads to rapid depolarization, and large influx of calcium and potassium. The intracellular calcium overload results in activation of excitotoxic glutamatergic transmission, nitric oxide (NO) synthase, caspase, xanthine oxidase and release of reactive oxygen species^15^ (Fig.).

Excess glutamate release leads to activation of phospholipases, phospholipid hydrolysis and arachidonic acid release, ultimately resulting in necrotic as well as apoptotic cell death^16^–^18^. Generation of free radicals, lipid peroxidation, inflammatory cascade and activation of immediate early genes such as *c-fos, c-jun*, leads to progressive ischaemic damage.

The generation of reactive oxygen species in the core area is less as compared to penumbra because there is negligible blood supply to core. The penumbra is still the viable tissue surrounding the core, and receives trivial amount of blood from collateral arteries^19^. This area is therefore, the target for drug intervention and has potential for recovery. If no quick and effective drug intervention is done, this further progresses to cellular energy failure, release of excitotoxic neurotransmitters and reactive oxygen species and finally, cellular death in the region.

## Mechanism of cell death

### 

#### Neurodegeneration

Neurodegeneration is described by the loss of neuronal functions and cell death^20^. Neuronal degeneration and cell death occurs through apoptotic as well as necrotic pathways^21^. Cell death is characterized by imbalance in cellular homeostasis, influx of calcium, mitochondrial dysfunction and generation of reactive free radicals^22^,^23^.

(*i*) Necrotic cell death - Energy depletion leads to release of excitatory amino acids (EAAs) such as glutamate^24^. Excessive release of glutamate activates α-amino-3-hydroxy-5-methlyl-4-isoxazolone-proprionic acid (AMPA) receptors which increase sodium influx^25^ and the N-methly-D-aspartate (NMDA) receptors which mediate the influx of calcium^26^. This increase in calcium overload results in activation of proteases such as caspases and matrix metalloproteases (MMP). The aftermath of this activation is increased proteolytic injury and ultimately cell death^27^. Many calcium dependent and calcium induced enzymes mediate intracellular calcium induced toxicity like NO synthase, cyclooxygenase, phospholipase A _2_ and calpain1^28^. Increased intracellular calcium activates NO synthase and results in release of NO^29^. The NO then combines with superoxide generated as by- product from cyclooxygenase, or xanthine oxidase to produce highly reactive peroxynitrite, that results in tissue destruction^30^. Cells are not capable of protecting themselves against excessive reactive oxygen species and ultimately die.

(*ii*) Apoptotic cell death - In apoptotic cell death there is transcription of immediate early genes such as *c-fos*, *c-jun* leading to caspase cascade resulting in increased cytokine levels within hours of initial injury. The released cytokines cause activation of cell surface receptors such as Fas receptor and tumour necrosis factor-alpha receptor (TNF- α) leading to apoptotic cell death^31^ ^32^. TNF- α stimulates the production of bcl-2 family protein, bid^33^. Bid activates bax, another bcl-2 family member and increases mitochondrial permeability, resulting in release of cytochrome c, a key component in apoptosis initiation. Cytochrome c forms a complex with apoptotic protease activating factor-1 (APAF -1) and procaspase -9, this complex causes cleavage of procaspase – 9 to caspase 9 and ultimately activation of other caspases including casapase-3^34^. Caspase-3 injury leads to irreversible DNA damage and cell death^35^. Generation of reactive oxygen species during cerebral ischaemia also activates process of apoptosis^36^ leading to activation of transcription factor p53 and caspases thus resulting in DNA damage^37^.

### Inflammation and ischaemia - role of microglia cells

The continued ischaemic injury to brain cells results in to a complex inflammatory cascade. It is characterized by infiltration of leukocytes mainly polymorphonuclear (PMN) cells, monocytes/macrophages lymphocytes and the activation of microglia which are the resident immune cells of the brain^38^. Astrocytes, neuronal support cells, also contribute to inflammation during insult^39^. Astrocytes under normal conditions perform several functions like, glutamate uptake, glutamate release and maintain cellular and ion homeostasis^40^. During cerebral injury, astrocytes undergo morphological changes and become activated^41^. Activated astrocytes release proinflammtory cytokines and chemokines thus results in initiation and progression of inflammation^39^.

In cerebral ischaemia, microglia, resident brain macrophages become activated and release detrimental neurotoxic mediators like proinflammtory cytokines, superoxide, nitric oxide (NO), TNF- α and proteases 42–44. Many of these mediators can inturn influence the microglia morphology and activate it in a paracrine and autocrine fashion^45^. Among the proinflammatory cytokines interleukin- 1 (IL-1) is most abundantly expressed in microglia cells^46^. IL-1 induces the expression of other cytokines such as IL-6 and TNF-α^47^ which contribute to the progression of ischaemic damage. The inhibition of this pathway of neuronal damage can be considered a logical option.

Other agents that have an important role in stroke injury are proteases. Tissue plasminogen activator, a potential neurotoxicant released by microglia cells is implicated in cerebral ischaemic injury^48^. Other proteases that play a leading role in disruption of blood brain barrier are matrix metalloproteases which contribute to secondary brain damage in cerebral ischaemia^49^. NO is a small molecule, secreted by microglia cells, acts directly on neurons as neurotoxin or indirectly by potentiating excitotoxic transmitters^50^. Inhibition of microglia activation in ischaemia may provide a novel target in management of stroke, the immunosuppressive agents, therefore, may serve the purpose to a greater extent.

### Is rtPA good enough in acute stroke?

Currently, recombinant tissue plasminogen activator (rtPA) is the only drug approved by US Food and Drug Administration for management of acute ischaemic stroke^51^. Several limitations are associated with rtPA including the narrow 3 h therapeutic window, increased risk of intracranial haemorrhage, generation of free oxygen radicals and recurrent stroke^5^,^51^. The rtPA has only clot busting activity and this activity is bothersome because it can lead to progression of secondary neuronal damage^52^.

For developing potent neuroprotective agents two main strategies are focused, clot lysis activity and safeguard of neurons subjected to ischaemic damage. rtPA is adequate for clot lysis, but there have been many unsuccessful attempts in developing effective neuroprotective drugs for management of stroke.

### Drugs under investigation for ischaemic stroke

Several studies conducted on drugs of different groups have exhibited neuroprotection in experimental models of cerebral ischaemia. One of these agents is endothelin antagonist (TAK-044), a reported anti-inflammmatory and antioxidant agent^53^. In oxygen-glucose deprivation model of cerebral ischaemia, TAK-044 showed significant improvement in percentage cell viability as compared to cells in hypoxic condition^54^. In rat model of focal cerebral ischaemia, TAK-044 significantly improved the neurological parameters, oxidative stress markers and infarct volume^53^. Several other agents have also shown potential of protecting the ischaemic cerebral injury by antioxidant mechanism. Agents like α-tocopherol^55^, trans-resveratrol^56^ and melatonin^57^ have shown significant improvement in neurobehavioural paradigms, infarct size and oxidative stress markers in experimental model of cerebral ischaemia.

The other group of drugs that showed promise is statins, 3-hydroxy-3-methyglutaryl (3- HMG)- CoA reductase inhibitors. Several studies have reported the beneficial effects of statins in experimental model of cerebral ischaemia^58^–^60^. The neuroprotective effects of statins are reported to be mediated by inhibiting inducible NO synthase thus resulting in inhibition of proinflammtory cytokines^60^. Beside these agents, recent studies have also shown neuroprotection by human umbilical cord cells^61^,^62^ in experimental models of cerebral ischaemia.

Recent findings have focused on drugs which can be given as stand alone or in combination with rtPA for management of stroke. One of these agents is imatinib, an anticancer drug. Imatinib is tyrosine kinase inhibitor used in treatment of chronic myeloid leukaemia. The rtPA neurotoxicity is thought to be mediated through platelet derived growth factor–CC (PDGF-CC)^63^. Recently Su *et al*^63^ have shown that administration of imatinib, PDGF-CC antagonist, one hour after vessel occlusion and 5 h later application of rtPA drastically reduced the infarct volume by 34 per cent and incidence of intracranial haemorrhage by 50 per cent as compared to rtPA alone in mouse model of stroke. One single administration of imatinib before the rtPA administration may provide an adequate neuroprotection in stroke patients, but this aspect needs to be validated in clinical settings. Till these agents are tested in clinical trials, no concrete conclusions can be drawn from animal models.

### Neuroprotection by immunosuppressive agents: a new dimension

As the ischaemic stroke has a multifactorial aetiopathology, and the mechanisms that are involved are activation of glutamate receptors, release of proinflammatory cytokines, TNF-α, NO, reactive oxygen radicals and proteases, the drugs having combinational neuroprotective and anti-inflammatory activity will be theoretically more effective in its management. Immunosuppressive agents have shown potential as neuroprotectants and anti-inflammatory agents in experimental models of cerebral ischaemia (Fig.). Drugs including steroids, immunophilin ligands and mycophenolate mofetil (MMF) have been in use as immunosuppressants since 1970s and these have been effectively used in avoiding organ rejection in transplant patients. Recent studies have reported their protective effect in microglial and neuronal cell cultures and in animal models of cerebral ischaemia^64^. Steroids, cyclosporine A, FK506, MMF and minocycline have been reported to have direct inhibitory effects on activation of microglia cells. This effect is mediated by inhibition of secretion of proinflammtory cytokines and NO from microglia cells^64^ (Table).

**Table:** Effects produced by immunosuppressive agents in experimental models of ischaemic stroke

Immunosuppressive drugs investigated	Experimental model	Effect produced	References
Steroids (Cortisol, corticosterone & dexamethasone) Dexamethasone	Isolated glial cells	Inhibition proliferation and activity of iNOS.	Chao *et al*, 1992^69^; Drew & Chavis, 2000^70^;
		Inhibition of release of TNF- α and IL-6.	Jacobsson *et al*, 2006^71^
		Suppression of production of MCP-1.	
	Permanent middle cerebral artery occlusion model in rats.	Reduction in expression of TNF- α.	Bertorelli *et al*, 1998^72^
	Permanent middle cerebral artery occlusion model in cats.	Beneficial effect on cerebral oedema.	Fenske *et al*, 1979^73^
	Occlusion of middle cerebral arteries in hyperglycaemic cat.	Reduce size of infarcts.	de Courten-Myers, 1994^74^
Cyclosporine A	Rat neuronal cells.	Inhibit caspase activation.	Capano *et al*, 2002^9^
	Human neuroblastoma cells.	Prevents apoptosis and enhance neurite outgrowth.	Sheehan *et al*, 2006^84^
	Transient forebrain ischaemia in rats.	Reduced brain oedema and infarct size.	Shiga *et al*, 1992^85^
	Hippocampal CA1 neurons.	Improvement in survival.	Uchino *et al*, 1995^86^; Miyata *et al*, 2001^87^
	Global ischaemia rat model.	Inhibit activation of microglia cells secreted proinflammatory substances.	Wakita *et al*, 1995^88^
Tacrolimus, FK506	Cortical cell against excitotoxic neuronal death.	Prevents dephosphorylation of nitric oxide synthase.	Dawson *et al*, 1993^92^
	Rodent model of focal ischaemia.	Neuroprotective effect.	Sharkey and Butcher, 1994^11^
	Transient global ischaemia model in gerbils.	Reduce delayed neuronal death mediated by inhibition of neuronal nitric oxide synthase.	Tokime *et al*, 1996^94^
	Primate model of cerebral ischaemia.	Improved neurological functions.	Furuichi *et al*, 2006^95^
Rapamycin	Microglial cells.	Inhibits expression of inducible nitric oxide synthase.	Lu *et al*, 2006^100^
	Traumatic injury model in mice.	Improved the functional recovery.	Erlich *et al*, 2007^101^
Mycophenolate Mofetil	Organotypic hippocampal slice cultures.	Attenuated neuronal damage induced by excitotoxic injury.	Dehghani *et al*, 2003^104^
	Mice model of amyotrophic lateral sclerosis.	Reduced microglial activation, improved stem cell survival and delayed the onset of neurological symptoms.	Yan *et al*, 2006^106^
Minocycline	Mixed SC and pure microglia cultures.	Inhibition of NMDA activated p38 MAPK, No and IL- 1β release.	Tikka *et al*, 2001^112^
	Global ischaemia in gerbils.	Inhibition of microglial cells.	Yrjanheikki *et al*, 1998^12^
	Focal ischaemia in rats.	Reduced infarct volume & Inhibition of microglial cells.	Yrjanheikki *et al*, 1999^13^

iNOS, inducible nitric oxide synthase; TNF- α, tumor necrosis factor-alpha; IL-6, interleukin- 1; MCP-1, monocyte chemoattractant protein-1; NMDA, *N*-methyl-D-aspartate; p38 MAPK, p38 mitogen-activated protein kinase; NO, nitric oxide

### Steroids in cerebral ischaemia: an unresolved issue

For many years corticosteroids have been used for the treatment of brain oedema^65^. Overwhelming evidence has reported the protective effects of corticosteroids, both *in vitro* as well as in a variety of animal models^66^,^67^.

*In vitro* studies using steroids have shown inhibition of both microglial cell proliferation and activity of NO synthase^68^ in the isolated microglial cells. Several investigators have reported that treatment of isolated microglial cells with steroid reduce the release of the proinflammatory substances^69^–^71^ and thus restricting the inflammation cascade and protecting neurons against the ischaemic insult. Treatment with dexamethasone on gerbil hippocampal regions diminished the ischaemia induced glutamate toxicity and consequently protected the neurons^10^. Dexamethasone has also been shown to suppress monocyte chemoattractant protein-1 (MCP-1) production via inhibition of jun-N-terminal kinase and p38 mitogen activated protein kinase in activated microglial cells^8^.

In *in vivo* studies, application of dexamethasone in permanent middle cerebral artery occlusion model in rats reduced the expression of TNF-α and decreased the inflammatory cascade^72^. Dexamethasone treatment in a model of permanent occlusion of middle cerebral artery in cats showed neuroprotective effects^73^. In an interesting experiment by de Courten – Myers *et al*^74^ 4 h occlusion of middle cerebral arteries in hyperglycaemic cats, and administration of high dose of corticosteroids 30 min after occlusion, significantly reduced the size of infarcts as compared to untreated group.

The mechanism of neuroprotection by corticosteroids is speculated to be either due to activation of membrane receptors on neurons by circulating steroids and hence leading to rapid changes in properties of exposed neuron or their modulation of nuclear transcription of various genes^67^. The beneficial effects of corticosteroids are largely due to preservation of neuronal structures and improvement in behavioural functions.

Interestingly in contrast to the experimental evidences, in the clinical trials, the steroids have not shown any beneficial effects. In a retrospective study on patients with history of ischaemic stroke, disability and mortality rates were worse in patients taking steroids as compared to those without steroid treatment^75^. There was no difference between the outcomes *i.e*., the disability and mortality as well as adverse effects between the two groups. In another study, patients with acute cerebral infarction were treated either with dexamethasone or placebo and the patients in steroid group fared worse than the placebo group^76^.

In summary, corticosteroids have shown favourable neuroprotective profile in experimental models of ischaemic stroke but failed to provide any significant beneficial effect in ischaemic patients. Thus, the use of corticosteroids as neuroprotective agents still remains controversial and there is a need to re-evaluate these in well planned clinical trials.

### How effective are immunophilin ligands in stroke?

A plethora of evidence is available that indicates the role of immunophilin/calcineurin in brain function and development^77^–^79^. High levels of immunophilins are expressed in brain and these are believed to be involved in neuronal apoptosis mechanisms^80^. Cyclosporin, tacrolimus/FK506 and rapamycin are immunophilin binding ligands used in avoiding rejection of transplanted organs.

**Cyclosporine A:** Cyclosporine A is an 11 amino acid cyclic peptide of fungal origin having effective immunosuppressive action. The mechanism of action of cyclosporine A involves binding to cyclophilin, an intracellular protein belonging to immunophillin family. Cyclosporine A and cyclophillin complex inhibits calcium/calmodulin dependent activation of calcineurin and via this pathway cyclosporine inhibits production of IL-2^81^ and gamma interferon and other lymphokines^82^. Cyclosporine A can also reduce the expression of an intracellular adhesion molecule and affect subsequent inflammation cascade^83^.

Cyclosporine A has shown antiapoptotic activity by inhibiting activation of caspase in rat neuronal cells^9^. It showed protective effect against apoptosis in human neuroblastoma cells by enhancing neurite outgrowth^84^. Cyclosporine A has shown to reduce brain oedema, infarct size and improved the survival of hippocampal CA1 neurons probably due to enhanced phosphorylation of cAMP response element binding (CREB) protein and increase in production of brain derived neurotrophic factor in experimental model of transient forebrain ischaemia in rats^85^–^87^. In the global ischaemia rat model, cyclosporine A showed beneficial effects by inhibiting the activation of microglia cells and thus providing protection against microglia secreted proinflammatory substances^88^. Till now cyclosporine A has not been tested in clinical trials for the treatment of CNS disorders but it has been tried in autoimmune disorders.

Cyclosporine A treatment in rats with experimental autoimmune myasthenia gravis has shown protective effects by reducing the levels of acetylcholine receptor antibodies by 50 per cent^89^. Mahattanakul *et al*^90^ have reported antinflammatory effect of cyclosporine A in patients suffering from chronic inflammatory demyelinating polyneuropathy. In this study, cyclosporine A treatment was successful in 3 out of 8 patients and the therapy with this agent did not show any serious side effects. In summary, cyclosporine A does have potential in neuroprotective strategy and needs to be evaluated experimentally and in clinical trials.

**Tacrolimus/FK506:** FK506 is an immunosuppressant introduced in 1990s and the mechanism of action is similar to that of cyclosporine A. FK506 binds with FK506 binding protein (FKBP), an immunophilin, and makes a complex which results in inhibition of downstream activation of nuclear factor of activated T-cells (NFAT), hence controlling relevant immunological genes^91^.

In *in vitro* studies, FK506 has shown protection against excitotoxic neuronal death in cortical cell cultures^92^. In another *in vitro* study, FK506 protected the cultured cortical neurons against oxygen glucose deprivation. The action was inhibited in the presence of an antibody against FKBP-12 suggesting that FK506 acts via FKBP-12^93^.

FK506 has been shown to provide protection in rodent model of focal ischaemia and this neuroprotection was thought to be mediated by immunophillins^11^, but in subsequent study done in gerbils in transient global ischaemia model, FK506 reduced delayed neuronal death and this effect was not mediated by inhibition of neuronal NO synthase, again binding to physiological target was anticipated^94^.

In primate model of cerebral ischaemia, FK506 showed a significant improvement in neurological deficits^95^. FK506 has been shown to have neuroprotective effects on cultured neurons and also in experimental models of ischaemic stroke, still a mechanistic pathway for its neuroprotective effect of tacrolimus needs to be explored.

**Rapamycin:** Rapamycin is a macrolide antibiotic developed as an antifungal agent but was later discovered to have potent immunosuppressive and anti-proliferative properties. Rapamycin is an immunophillin ligand that structurally resembles FK506. Unlike FK506 and cyclosporine A, rapamycin binds to mammalian targets of rapamycin (mTOR) and prevents phosphorylation of p70S6K, 4EBP1 and other proteins involved in transcription, translation and cell cycle control^96^. Rapamycin also antagonizes the anti-apoptotic signals mediated by mTOR^97^, and induces autophagy and maintains normal cell metabolism^98^.

Literature is available stating the benefitting effects of rapamycin in *in vitro* and *in vitro* models of cerebral ischaemia. An *in vitro* study reported that application of rapamycin at different concentrations did not affect the bioelectrical activity and evoked field excitatory post-synaptic potentials magnitude in hippocampal slices^99^ suggesting an alternative to calcineurin inhibitors in events of neurotoxicity. Rapamycin treatment inhibits the hypoxia induced expression of inducible NO synthase in microglial cells^100^. In mice after experimental traumatic brain injury, rapamycin significantly improved the functional recovery^101^. Anti-depressant like effects of rapamycin in rodents have also been reported suggesting a role in treatment of affective disorders^102^. Effects of rapamycin in experimental models of cerebral ischaemia have not been systematically investigated. More studies have to be undertaken to fully acknowledge the neuroprotective significance of rapamycin.

### Does mycophenolate mofetil (MMF) have any potential as neuroprotectant?

MMF is a prodrug converted into active mycophenolic acid in body and inhibits inosine mono phosphate dehydrogenase, a critical enzyme required in *de novo* purine biosynthesis. Inhibition of this enzyme leads to reduction in purine nucleotides and inhibition of cellular proliferation. MMF suppresses the monocytic production of both pro-inflammatory cytokines and microglial mitogen granulocyte macrophage colony stimulating factor^103^. It has also shown to prevent microglial activation as well as reduce the neuronal damage induced by excitotoxic injury in the *in vitro* model of stroke^104^. The microglial activation is an important pathway of proinflammatory cytokines generation, nitric oxide production and activation and release of proteases, which leads to neuronal damage^105^.

Studies on the effects of MMF in animal models of CNS disorders are sparse. In mice model of amyotrophic lateral sclerosis, MMF treatment reduced microglial activation, improved stem cell survival and delayed the onset of neurological symptoms^106^. MMF has also shown protection against experimental autoimmune myasthenia gravis in rats^107^and has also been used against inflammatory myopathy and chronic inflammatory demylelinating polyradiculoneuropathy^108^. Thus MMF is an interesting candidate but its use in ischaemic brain diseases needs further exploration.

### Effects of minocycline - an approach to ischaemic damages?

Minocycline is semisynthetic tetracycline derivative, which also possesses anti-inflammatory properties that are entirely distinct from its antimicrobial action. Studies have shown its usefulness in management of rheumatoid arthritis and osteoarthritis^109^. Minocycline has been recognized to have numerous pharmacological effects including ability to inhibit MMP, superoxide production and iNOS expression in human cartilage and murine macrophages^110^,^111^. Minocycline has been reported to penetrate blood brain barrier and provide neuroprotection in global ischaemia in gerbils^12^ and focal ischaemia in rats^13^. In these studies the neuroprotection by minocycline was associated with reduced activation of microglia and also inhibition of induction of IL-1b-converting enzyme (ICE) mRNA which was expressed mostly in microglia. In nanomolar concentrations it protected neurons in mixed spinal cord (SC) as well as pure microglia cultures against NMDA excititoxicity^112^. NMDA activated p38 mitogen-activated protein kinase (p38 MAPK), NO and IL- 1β release were effectively inhibited by minocycline in mixed spinal cord and pure microglia cultures^113^. Thus it indicates the direct effect of monocycline on microglia activation and proliferation.

Minocycline also demonstrated delayed mortality in transgenic R 6/2 model of Huntington disease by inhibiting caspase 1 and 3 expression as well as iNOS activity^114^. Minocycline treatment exhibited protection in MPTP (1-methyl-4-phenyl-1, 2, 3, 6-tetrahydropyridine) model of Parkinson’s disease, this neuroprotection was mediated by reduction in expression of inducible NO synthase (iNOS) and caspase -1 expression^115^. Recently a placebo controlled open label clinical trial of minocycline in acute stroke has shown significant beneficial effects^116^. To the best of our knowledge three clinical trials are under investigation to evaluate the effects of minocycline in acute ischaemic stroke^117^. In one study, safe and well tolerated doses of minocycline are being evaluated whereas in other study minocycline, enoxaparin or combination of both will be studied on acute ischaemic stroke patients. The effect of minocycline on neurological deficits and functional outcomes in acute ischaemic stroke patients will be assessed in the other enlisted clinical trial^117^.

## Conclusion

Cerebral ischaemia is a multifactorial disorder which includes a number of pathways for progression of injury to brain cells. Activation of glutamate receptors, release of NO, proteases and generation of free radicals are important mechanisms in ischaemia. Inflammation is considered a major component in disseminating the detrimental effects of cerebral ischaemia. Circumscription of inflammatory cascade is an integral part in ameliorating the harmful consequences in cerebral ischaemia. In this respect, immunosuppressive agents can prove to be valuable drugs in attenuating CNS damage following ischaemia.

Immunosuppressive agents have been shown to undermine the CNS damage following ischaemia by improved neuronal survival and inhibition of mediators of inflammation in various experimental models. *In vitro* experiments using immunosuppressive agents have demonstrated the blockade of excitotoxic insult induced by NMDA as well as inhibition of microglia cells, thus inhibiting inflammatory cascade. *In vivo*, these have been shown to improve the neurological functions as seen by neurological assessment paradigms and reductions in infarct volume.

More research is required before any definitive conclusion is derived for immunosuppressive agents as neuroprotectants. Minocycline is the only drug which is being evaluated in clinical trials. An important issue that needs to be addressed is: can there be a differential dose that causes neuroprotective effect than that required to cause immunosuppression? If these drugs exhibit neuroprotection at much lower doses than that required for preventing organ transplant rejection, it may be a potential addition to the existing therapeutic armamentarium for ischaemic stroke. This will require well designed clinical trials to evaluate short as well as long term clinical outcomes *vis-a-vis* side effect profile of each drug and translate it into improvement in quality of life of stroke patients.
